# Effect of the Fermentation Broth of the Mixture of *Pueraria lobata*, *Lonicera japonica*, and *Crataegus pinnatifida* by *Lactobacillus rhamnosus* 217-1 on Liver Health and Intestinal Flora in Mice With Alcoholic Liver Disease Induced by Liquor

**DOI:** 10.3389/fmicb.2021.722171

**Published:** 2021-08-17

**Authors:** Ting Wang, Zhe Wang, Zhipeng Yang, Xin Cui, Liang Yan, Zhenshang Xu, Xinli Liu

**Affiliations:** ^1^State Key Laboratory of Biobased Material and Green Papermaking, Qilu University of Technology, Shandong Academy of Science, Jinan, China; ^2^Shandong Provincial Key Laboratory of Microbial Engineering, Department of Bioengineering, Qilu University of Technology, Shandong Academy of Science, Jinan, China

**Keywords:** *Lactobacillus rhamnosus*, *Pueraria lobata*, *Lonicera japonica*, *Crataegus pinnatifida*, alcoholic liver disease, intestinal flora, gut-liver axis

## Abstract

In this work, we discovered a new fermentation broth that can prevent and regulate alcoholic liver disease (ALD) and intestinal flora, which fermented the mixture of *Pueraria lobata*, *Lonicera japonica*, and *Crataegus pinnatifida* by *Lactobacillus rhamnosus* 217-1. The contents of polyphenols, puerarin, total isoflavones, and amino acids were significantly increased. Animal experiments showed that the fermentation broth could improve the liver indexes of ALD mice model, increase the activity of superoxide dismutase and glutathione in liver tissue, and reduce the level of malondialdehyde (MDA). Furthermore, the fermentation broth can reduce the levels of serum lipopolysaccharide (LPS), inflammatory factors interleukin-6 (IL-6), and tumor necrosis factor-α (TNF-α). Importantly, intestinal flora analysis showed that the fermentation broth could increase the abundance of *Lactobacillales* and reduce the production of Gram-negative bacteria, thereby reducing the abnormal increase in bacterial diversity caused by alcohol. In conclusion, we may have discovered a new functional food raw material with great application potential. The above findings indicate that the fermentation broth can actively regulate the intestinal flora and improve liver inflammation. The underlying mechanism might be that the fermentation broth could enhance intestinal permeability and reduce the inflammatory signals and LPS transmitted through the gut-liver axis, thereby reducing the oxidative stress and inflammation of the liver caused by alcohol.

## Introduction

Due to the injury effects and the lack of effective treatment strategies, alcoholic liver disease (ALD) has become a major public health problem (Li et al., 2019). ALD can be divided into many types, including liver steatosis, steatohepatitis, acute alcoholic steatohepatitis, alcoholic fibrosis, cirrhosis, and hepatocellular carcinoma (HCC; Wan et al., 2020). At present, studies of ALD mainly focus on oxidative stress, inflammatory responses, lipid peroxidation, endogenous endotoxins, and nutritional imbalances related to alcohol metabolism (Kong et al., 2019; Ran et al., 2020). Based on the above researches, some treatments for ALD have been developed, such as polyene phosphatidylcholine, phosphodiesterase inhibitors, and glucocorticoids. Except for glucocorticoids in the settings of alcohol hepatitis with Maddrey Discrimination Function more than 32, other drugs have not been proved efficacy (Mathurin and Lucey, 2012; Addolorato et al., 2020). However, the drug resistance and infection risk usually increase after long-term use (Gao and Bataller, 2011). Up to now, no specific treatment for this disease has been approved by the U.S. Food and Drug Administration (FDA; Singal et al., 2018; Li et al., 2021). Therefore, the gold standard of treatment for ALD is total alcohol abstinence, and it is very urgent to find new ways to prevent or alleviate ALD.

*Pueraria lobata* has been widely accepted by consumers all over the world owing to its health effects (Wang et al., 2008). Animal experiments have shown that the isoflavones (including puerarin, daidzin, and daidzein) extracted from *P. lobata* inhibit alcohol intake and eliminate the occurrence of alcohol withdrawal symptoms (Kang et al., 2015). Simultaneously, isoflavones improve the antioxidant capacity and reduce oxidative damage of livers (Lee et al., 2001). However, the bioavailability of isoflavone aglycone is much higher, which can be transformed from isoflavones by the fermentation of microorganism (Izumi et al., 2000). As medicinal and edible materials, *Lonicera japonica* and *Crataegus pinnatifida* have been cultivated for a long time in China. They are traditionally used as food additives in tea, beverages, wine, and yogurt (Shang et al., 2011; Zhang et al., 2018). *Lonicera japonica* has a high content of polyphenols, such as chlorogenic acid (CGA), which is known for its effective antioxidant and free radical scavenging activities. The bioactive components of *C. pinnatifida* contain flavonoids, polyphenols, steroids, organic acids, and triterpenoids (Wu et al., 2014). Several recent studies have shown that *L. japonica* and *C. pinnatifida* significantly reduce liver tissue damage and exhibit hepatoprotective effects in ALD rat models (Ge et al., 2019; Li et al., 2020).

Alcohol consumption leads to quantitative and qualitative dysbiosis in the intestinal microbiota of rodents and humans. Dysbiosis in the intestinal microbiota may contribute to the pathogenesis of liver disease by altering intestinal barrier function. The increase in intestinal permeability allows pro-inflammatory/pathogenic microbial products including endotoxins (such as LPS and peptidoglycan) to be transported from the intestinal lumen to the liver through the portal vein, causing liver inflammation. Studies have shown that *Lactobacillus rhamnosus* GG (LGG) alone or rich in oat fiber can effectively improve “leaky intestine” and hepatitis (Forsyth et al., 2009). Administration of LGG has protective effects on ethanol-induced liver injury, circulating liver enzymes [such as alanine aminotransferase (ALT) and aspartate aminotransferase (AST)], and endotoxemia in Wistar rats (Nanji et al., 1994). Transformation of intestinal flora may be a therapeutic target for intestinal barrier dysfunction and ALD. These findings suggest that intestinal flora plays a role in alleviating alcohol-induced malnutrition and liver injury. Besides, the regulation of intestinal microflora may be a feasible treatment strategy to prevent or normalize alcohol-induced malnutrition and may have beneficial effects on alcohol-induced liver injury and other inflammation-mediated diseases caused by long-term drinking.

Lactic acid bacteria (LAB) as starter are used in a variety of fermentation processes. The quality of the product can be improved by the fermentation of LAB. On the one hand, the sugar content or anti-nutrients in the substrate is reduced, and beneficial molecules, such as bioactive peptides, short-chain fatty acids, or polysaccharides are produced. On the other hand, some phenolic compounds are converted into molecules with additional biological value (Septembre-Malaterre et al., 2018). Therefore, LAB fermentation enhances the concentration of active substances and the antioxidant capacity of fermentation broth (Sanjukta et al., 2015; Yan et al., 2019). Furthermore, several probiotics have been tested in rodent models and in some human trials used to slow down the process of ALD (Cassard and Ciocan, 2017). VSL#3 is a mixture of eight probiotic strains (mainly *Lactobacillus* and *Bifidobacterium*), also improved liver lesions in humans and rodents with ALD (Cfederico, 2005; Bing et al., 2013). *Lactobacillus plantarum* KLDS1.0344 and *L. acidophilus* KLDS1.0901 mixture may indirectly inhibit the TLR/NF-κB signaling pathway to inhibit liver inflammation by improving the permeability of the intestinal epithelium and regulating the intestinal flora (Li et al., 2021).

Considering the liver protection role of the active substances in the above-mentioned plant raw materials, and the safety and function of probiotics, the medicinal plants including *P. lobata*, *L. japonica*, and *C. pinnatifida* were fermented by *L. rhamnosus* 217-1 in the present study. Furthermore, the changes in active ingredients of fermented mixture were determined, and the effect of the fermentation broth on the ALD model in mice was explored. The obtained results provide a basis for the development of new treatments for ALD.

## Materials and Methods

### Strain, Materials, and Chemicals

The *P. lobata*, *L. japonica*, and *C. pinnatifida* were purchased from Beijing tong ren tang international Co., Ltd. (Beijing, China). The strain used in this experiment was preserved in our laboratory and showed good fermentation ability in the previous experiment (data not shown). 2,2-diphenyl-1-picrylhydrazyl (DPPH) and Folin–Ciocalteu reagents were obtained from Aladdin Company (Shanghai, China). All other chemicals were procured from Sinopharm Chemical Reagent Co., Ltd. (Shanghai, China).

### Preparation of Fermentation Broth

*Lactobacillus rhamnosus* 217-1 was routinely cultivated in MRS liquid medium overnight at 37°C. *Pueraria lobata*, *L. japonica*, and *C. pinnatifida* were ground into powder. The fermentation medium was composed of 20g glucose, 1.25g whey protein powder, 50g *P. lobata*, 25g *L. japonica*, 25g *C. pinnatifida*, and 500ml distilled water. After autoclaving at 110°C for 20min, *L. rhamnosus* 217-1 was inoculated into the fermentation medium and then cultured at 37°C statically for 21days. The control experiment was conducted without the addition of *L. rhamnosus* 217-1. Fermentation broth samples were collected on day 0, day 7, day 14, and day 21, respectively. They were centrifuged at 4,000rpm/min for 10min, and the supernatants were collected and stored at 4°C for further analysis.

### Determination of Active Substance Concentration in Fermentation Broth

#### Total Polyphenol Compounds Concentration

Total polyphenol concentrations were determined by using the Folin–Ciocalteu’s reagent as described by Huang et al. (2017). Briefly, 50μl of sample solution was mixed with 2.0ml of 2% Na_2_CO_3_ and incubated at room temperature for 2min. Subsequently, 100μl of 50% Folin–Ciocalteu’s phenol reagent was added and mixed thoroughly, and then incubated at room temperature for 30min in the dark. Absorbance was measured at 720nm, and polyphenol concentrations were expressed as gallic acid equivalents (mg of GAE/ml of sample).

#### Total Puerarin Concentration

The supernatant was filtered through a polytetrafluoroethylene filter (0.45μm) for high performance liquid chromatography (HPLC) analysis. The chromatography column was ZORBAX C18 (4.6mm × 250mm, 5μm, Agilent, United States). The mobile phase was composed of methanol, acetic acid, and water (25: 1.08: 73.92), and the flow rate was 0.6ml/min. The puerarin was detected at the column temperature of 25°C and the wavelength of 247nm.

#### Total Isoflavone Concentration

The total isoflavone concentration (expressed in puerarin equivalent) was measured by using UV-2012 UV–visible spectrophotometer (Unico, Wisconsin, United States) at the wavelength of 250nm. The puerarin with different concentrations was used as a standard to determine the content of total isoflavone.

#### Total Amino Acid Concentration

In order to detect the types and concentrations of amino acids in the fermentation broth, the samples were filtered with polytetrafluoroethylene filter and analyzed by using Hitachi l-8,900 automatic amino acid analyzer (Tokyo, Japan).

### Determination of Antioxidant Activity

2,2-diphenyl-1-picrylhydrazyl radical scavenging activity was measured according to the method described by Huang et al. (2017). In brief, a 200μl sample was mixed with 2.0ml of 0.16mM of DPPH solution sufficiently. The mixture was incubated at room temperature for 30min in the dark. Subsequently, the absorbance was measured at 517nm. Vitamin C (10mg/l) was used as a positive control. The percentage scavenging activity was determined using the following equation:

$$DPPHradicalscavengingactivity\;\left(\%\right)=\left[1-\frac{Ai-Ai0}{A0}\right]\times 100$$

where Ai was the absorbance of sample with DPPH, Ai0 was the absorbance of sample without DPPH, and A0 was the absorbance of DPPH without sample.

Finally, according to the standard curve of DPPH radical scavenging rate, the IC50 (median inhibition concentration) value of the sample was obtained.

### Animal Experiment

Male C57BL/6J Kunming mice (6-week old) were purchased from Jinan Pengyue Experimental Animal Breeding Co., Ltd. (Shandong, China) with the number of SCXK (Lu) 2014-0007. All animal experiments were approved by the Experimental Animal Ethics Review Committee of Yantai Raphael Biotechnology Co., Ltd. [Permit Number: SCXK (Lu) 2017-0026]. After 1week of acclimatization, 42 mice were randomly divided into three groups: the normal group (CK, *n*=14), the model group (ET, *n*=14), and the test group (ET-J, *n*=14). The experimental method is based on Ran et al. (2020) with some modifications. After grouping, the mice were given intragastric administration at a dose of 0.1ml/10g animal body weight for 20days. Every morning during this period, the 0.9% normal saline was administered to the CK group, while equal amounts of white spirit (56°) were administered to the other groups. Based on the results of the previous part of the experiment, the fermentation supernatant of day 21 was chosen to gavage the mice. After 8h of feeding the liquor, the fermentation broth supernatant was given to each mice of the ET-J group by gavage, and 0.9% normal saline was given to the other two groups.

The mice were treated with fasting and water deprivation for 12h after the final dose, followed by anesthetization using light ether. The blood was collected from eye veins and then centrifuged at 3,500r/min at 4°C for 10min to obtain the serum. Subsequently, the mice were sacrificed promptly by cervical vertebra dislocation and dissected to take the liver. After washing with ice normal saline solution and removing water on the liver surface, the weight of the liver was examined to calculate the liver index.

$$Liverindex\left(\%\right)=\frac{Liverweight}{Bodyweight}\times 100\%$$

### Biochemical Analysis

The ALT and AST contained in serum were determined using corresponding enzyme-linked immunosorbent assay (ELISA) kits (Nanjing Jiancheng Institute of Bioengineering, Nanjing, China). Appropriate amounts of normal saline were added to the liver tissue, and then the mixture was homogenated for 2min. After centrifuged at 3,500r/min at 4°C for 15min, the supernatants were taken for analysis. Tumor necrosis factor-ɑ (TNF-ɑ), interleukin-6 (IL-6), malondialdehyde (MDA), and superoxide dismutase (SOD) were measured according to the instructions of the ELISA kits (Quanzhou Konodi Biotechnology Co., Ltd., Fujian, China). The glutathione (GSH) content was determined by using the GSH assay kit (Nanjing Jiangcheng Bioengineering, Institute Nanjing, China).

### Fecal Microbial Community Analysis

Total bacterial DNA was extracted from fecal samples using a Stool DNA Kit (MAGEN, Guangzhou, China) following the manufacturer’s procedures. Sequencing of intestinal flora was completed in Guangzhou Gene Denovo Biotechnology Co., Ltd. (Guangzhou, China).

### Data Analysis

All the experiments were conducted in triplicate, and the results were expressed as mean±SD. Significant analysis between different groups was performed by least significant difference (LSD) test using SPSS 26.0 software (SPSS Inc., Chicago, IL). All statements of significance were based on probability of *p*<0.05.

## Results

### Changes in Active Substance Content and Antioxidant Activity During Fermentation

**Figure 1:**
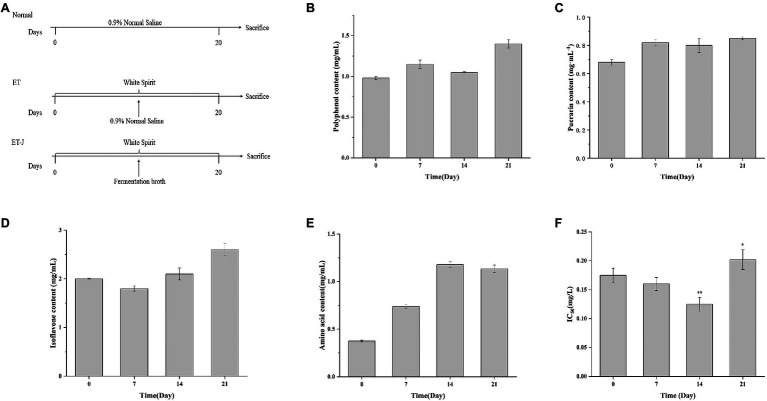
**(A)** Schematic diagrams showing the three treatment strategies, **(B)** total polyphenol concentration, **(C)** total puerarin concentration, **(D)** total isoflavone concentration, **(E)** total amino acid concentration, and **(F)** IC_50_ of fermentation broth during *Lactobacillus rhamnosus* 217-1 fermentation. Results are expressed as the mean±SD (*n*=3). ^*^p<0.05 vs. 0day; ^**^p<0.01 vs. 0day.

The changes of polyphenols, puerarin, isoflavones, and amino acids in the broth during the fermentation of *L. rhamnose* 217-1 are illustrated in Figure 1. The initial concentration of polyphenols, puerarin, and isoflavones was 0.954, 0.668, and 2mg/ml, respectively. After 21days of fermentation, the final concentration of these active substances was 1.4, 0.846, and 2.65mg/ml, respectively (Figures 1B–D). Through the fermentation of *L. rhamnose* 217-1, their content increased by 46.8, 26.6, and 32.5%, respectively. The concentration of total amino acids in the fermentation broth increased rapidly at first and then stabilized gradually. On the 14th day, the highest concentration of total amino acid was 1.178mg/ml, which was 214% higher than that of 0.375mg/ml before fermentation (Figure 1E). The detailed amino acid composition was determined and shown in Table 1.

**Table 1:** Species and composition of amino acids in fermentation broth.

	Time (day)			
	0	7	14	21
Types of amino acids	14	15	15	15
Types of essential amino acids	7	7	7	7
Ratio of essential amino acids (%)	40.6	29.1	23.2	31.4
The first six amino acids	Arginine	Arginine	Arginine	Arginine
	Threonine	Glycine	Glycine	Glycine
	Glutamate	Glutamate	Alanine	Tyrosine
	Aspartic acid	Threonine	Glutamate	Alanine
	Alanine	Leucine	Phenylalanine	Phenylalanine

The antioxidant capacity of the fermentation broth was tested by using the DPPH method. As shown in Figure 1F, the IC_50_ value of the fermentation broth decreased first and then increased during the fermentation process. The lowest value was observed on the 14th day with an IC_50_ of 0.1215, which was 30.7% lower than 0.1752 on day 0. With the extension of fermentation time, the antioxidant capacity of the fermentation broth decreased, and the IC_50_ value increased. This might be due to the consumption of other antioxidants besides puerarin, isoflavones, and polyphenols.

### Effects on Liver Index, ALT, and AST

**Figure 2:**
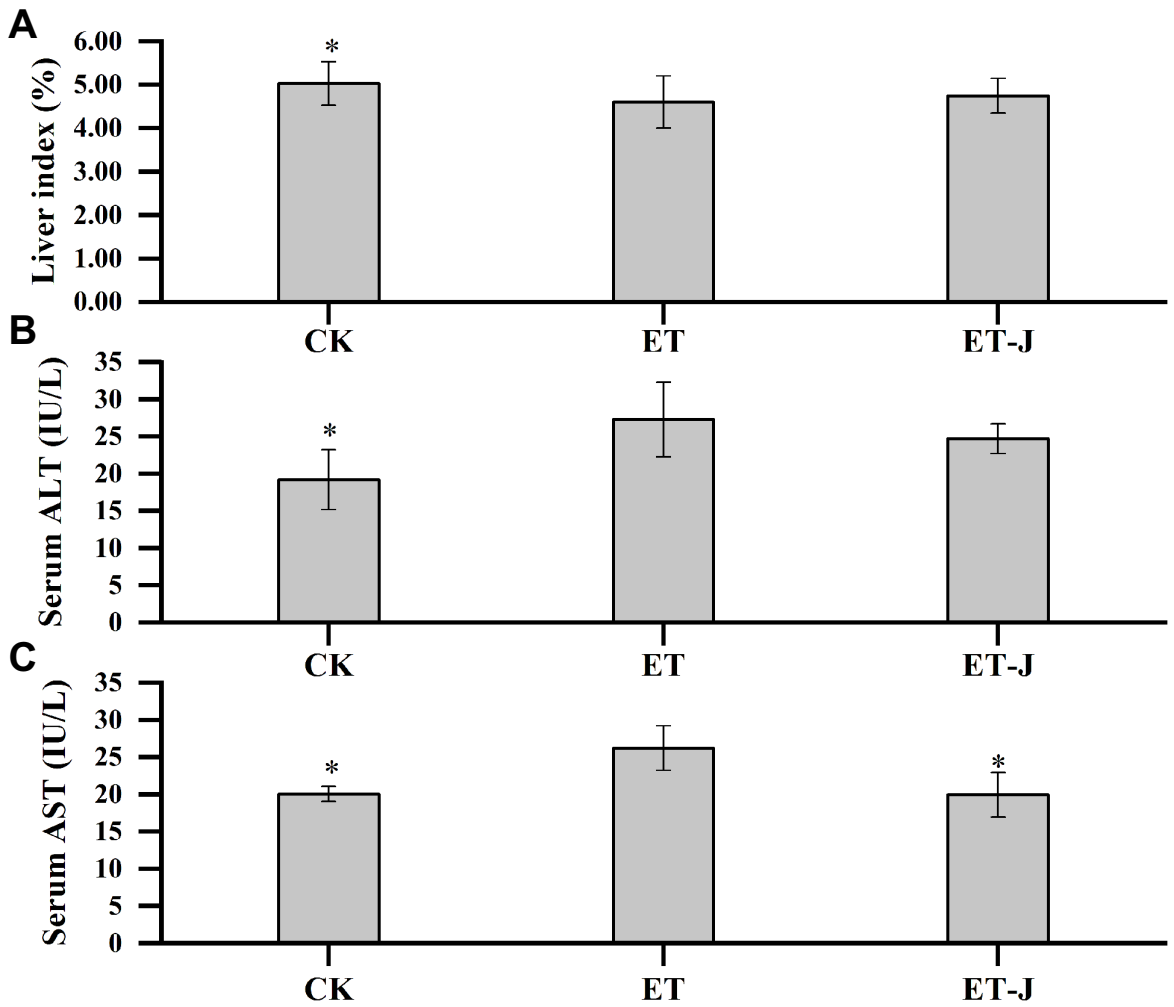
Effect of fermentation broth on **(A)** liver index and activity of **(B)** alanine aminotransferase (ALT) and **(C)** aspartate aminotransferase (AST) in serum. CK, mice were administrated with 0.9% normal saline; ET, mice were administrated with liquor; and ET-J, mice were administrated with liquor and fermentation broth (0.1ml/10g). Results are expressed as the mean±SD (*n*=14). ^*^p<0.05 vs. ET.

The effect of fermentation broth on the liver index of mice was investigated. As shown in Figure 2, the liver index of the ET group was significantly lower than that of the CK group. This result indicated alcohol caused severe damage to the liver of mice, leading to the phenomenon of liver atrophy and failure. However, the liver index of the ET-J group was close to the CK group, suggesting the fermentation broth had a relieving effect on the liver’s alcohol damage.

The ALT and AST levels were further measured. As shown in Figures 2B,C, alcohol significantly increased the ALT and AST levels of the ET group (*p*<0.05), while the level of AST in the ET-J group was effectively reduced (*p*<0.05). Although the level of ALT was also decreased without a significant difference. Apparently, the AST level of the ET-J group restored the average level. These results showed that the fermentation broth had good effects on protection and reduction of the pathological damage of liver caused by alcohol.

### Effect on Oxidative Stress in Mice Liver

**Figure 3:**
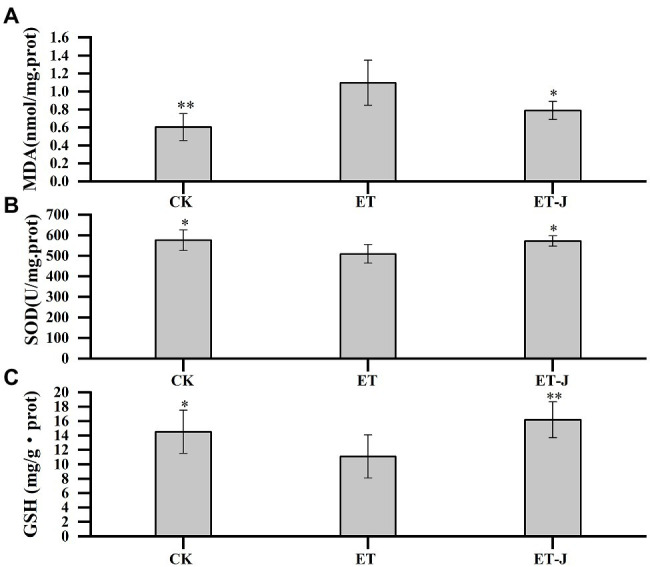
Effect of fermentation broth on oxidative stress parameters of liver in alcoholic liver disease (ALD) mouse model, including **(A)** malondialdehyde (MDA), **(B)** superoxide dismutase (SOD), and **(C)** glutathione (GSH). CK, mice were administrated with 0.9% normal saline; ET, mice were administrated with liquor; and ET-J, mice were administrated with liquor and fermentation broth (0.1ml/10g). Results are expressed as the mean±SD (*n*=14). ^*^p<0.05 vs. ET; ^**^p<0.01 vs. ET.

To investigate the effect of fermentation broth on liver oxidative stress, the MDA, SOD, and GSH in liver tissue were determined. As shown in Figure 3A, the MDA level of liver in the ET group was significantly increased (*p*<0.01). Meanwhile, SOD activity and GSH concentration were significantly decreased (*p*<0.05; Figures 3B,C). These results showed that the lipid peroxidation reaction was serious, which was resulted from the weakening of free radical scavenging ability. Compared with the ET group, the fermentation broth significantly decreased MDA level by 28.1% (*p*<0.05), and increased SOD activity and GSH content by 12.2 and 46.2% (*p*<0.05; *p*<0.01), respectively.

### Effect on Inflammatory Cytokines

**Figure 4:**
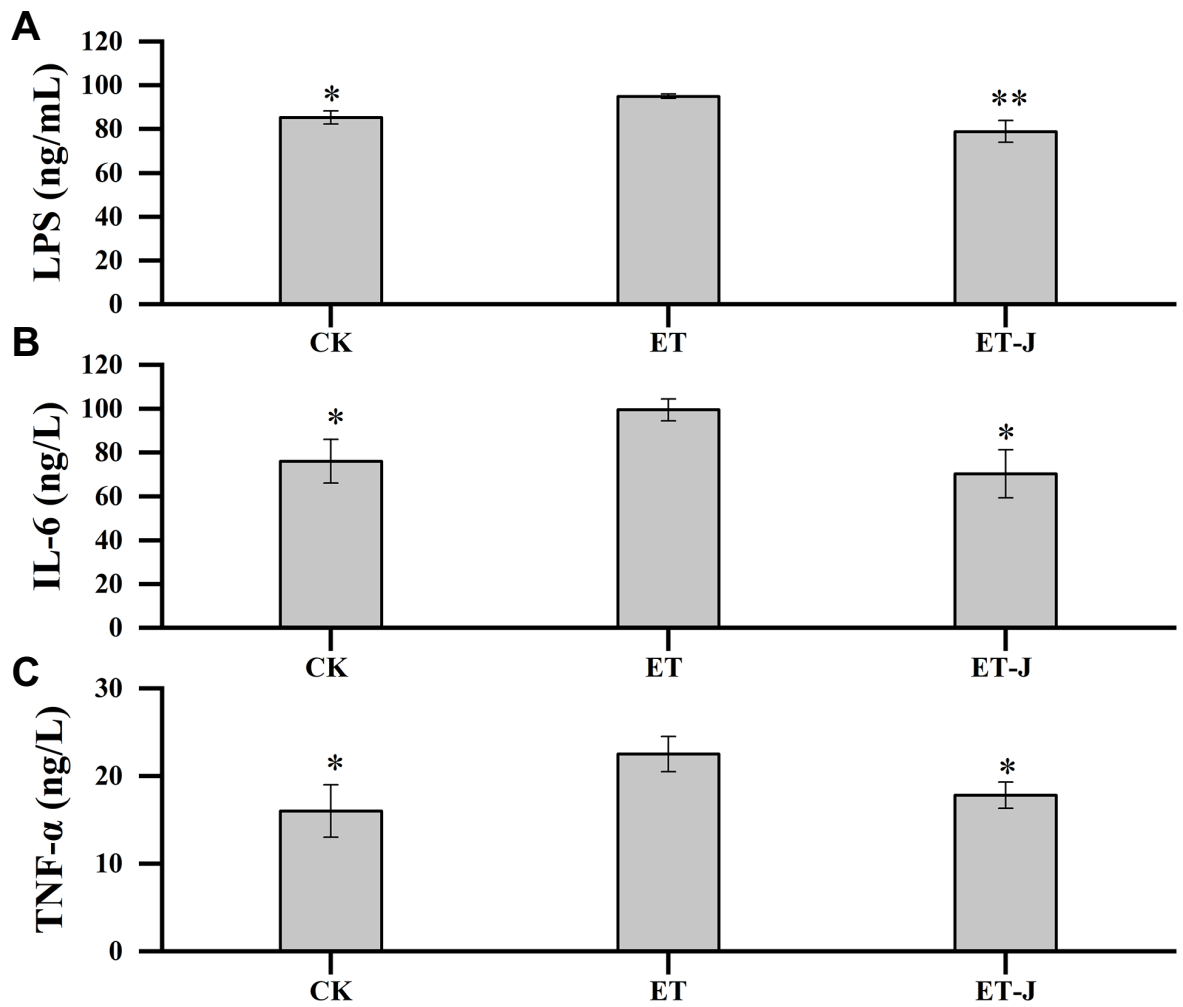
Effects of fermentation broth on the contents of **(A)** lipopolysaccharide (LPS) and **(B)** inflammatory factors interleukin-6 (IL-6), and **(C)** Tumor necrosis factor-α (TNF-α) in serum of ALD model mice. CK, mice were administrated with 0.9% normal saline; ET, mice were administrated with liquor; and ET-J, mice were administrated with liquor and fermentation broth (0.1ml/10g). Results are expressed as the mean±SD (*n*=14). ^*^p<0.05 vs. ET; ^**^p<0.01 vs. ET.

To investigate the effect of fermentation broth on liver inflammation, the LPS, IL-6, and TNF-ɑ in mice’s serum were detected. As shown in Figure 4, the LPS, IL-6, and TNF-ɑ of the ET group were significantly higher than those of the CK group (*p*<0.05). Compared with the ET group, the fermentation broth significantly decreased the concentration of LPS, IL-6, and TNF-ɑ. Their content was reduced by 16.9% (*p*<0.01), 29.4% (*p*<0.05), and 20.9% (*p*<0.05), respectively.

### Effect of Fermentation Broth on Intestinal Flora of ALD Mouse Model

Nakamoto et al. (2017) found that the gut-liver axis plays a pivotal role in regulating liver immune activation and tolerance in the recovery phase after acute liver injury. Therefore, we sequenced the microorganisms in the feces of three groups of mice and then analyzed the changes of intestinal flora in mice to explore the mechanism of fermentation broth intervention in ALD.

**Figure 5:**
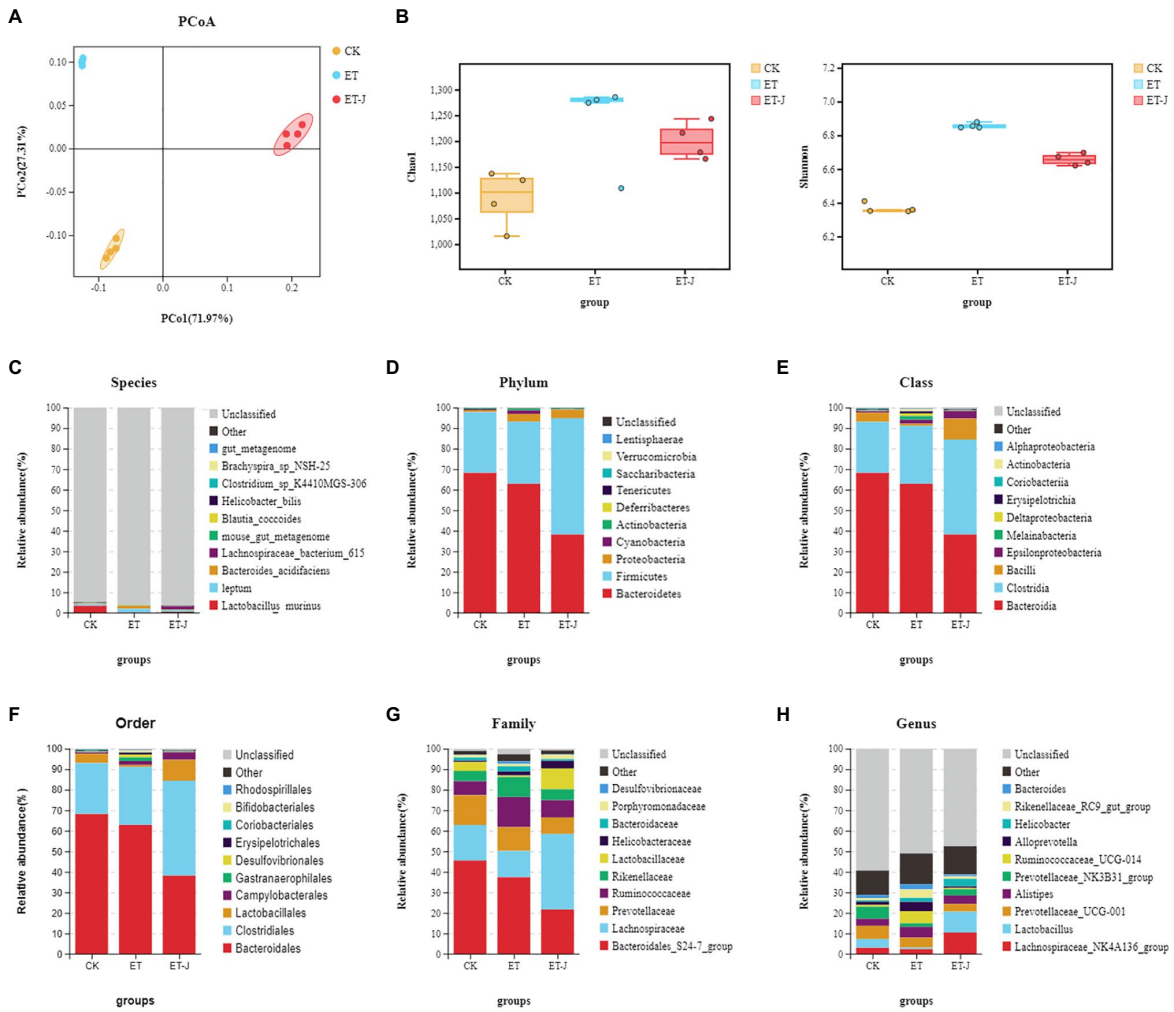
The fermentation broth modulated the composition of the gut microbiota. **(A)** PCoA analysis among three groups based on weighted UniFrac distances. Each plot represents one sample. **(B)** The α-diversity of the intestinal flora indicated by the Shannon index and the Chao index (*n*=14). **(C)** Species-; **(D)** Phylum-; **(E)** Class-; **(F)** Order-; **(G)** Family-; and **(H)** Genus-level taxonomic distributions of microbial communities in cecal contents determined by 16S DNA sequencing.

The α diversity Shannon index and Chao index (Figure 5B) showed that ALD induced by alcohol could increase the diversity of intestinal flora in mice, which is consistent with the research results of Li et al. (2021). And the fermentation broth could regulate the recovery of the normal level of intestinal flora in mice. Then, we performed a weighted UniFrac PCoA analysis to study the differences in species complexity and structural changes (β diversity) of intestinal microbial communities. The results revealed significantly different β diversity among the three groups (Figure 5A). This indicated that the fermentation broth could alleviate the imbalance of intestinal flora induced by alcohol.

Next, we evaluated the relative abundances of the main taxonomic groups of the intestinal flora and analyzed the structures of the intestinal flora, the top most abundant taxa at the species to genus levels are shown in Figures 5C–H. In all the mice, the structure of the intestinal flora was mainly composed of *Bacteroidetes* and *Firmicutes*, accounting for >90% of all bacteria. At the phylum level, compared with the CK group, alcohol caused the decrease in the abundance of *Bacteroides* in the ET group, the increase in the abundance of Firmicutes, and the continuous decrease in fermentation broth significantly strengthened this change. At the family level, the relative abundance of *Lactobaciliaceae* in the ET-J group increased relative to ET (*p*<0.05), while the relative abundance of *Ruminococcaceae* and *Rikenellaceae* in the bacterial family decreased, but the difference was not significant. At the genus level, the abundance of *Lachnospiraceae* and *Lactobacillus* in the fecal flora of the ET-J group was significantly higher than that of the ET group and CK group (*p*<0.05).

**Figure 6:**
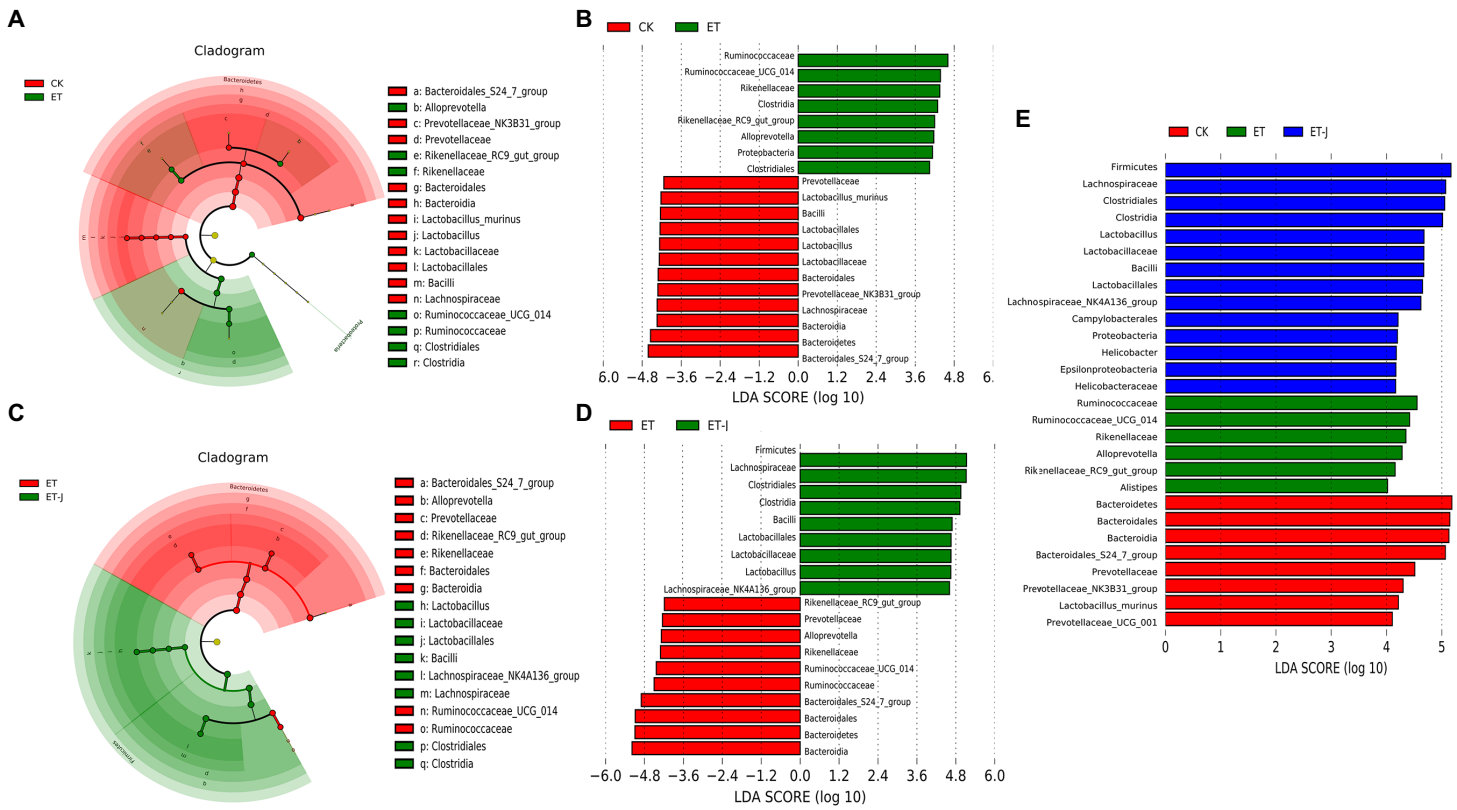
The fermentation broth reshaped the gut microbiota community. **(A)** LEfSe cladogram represents the taxa enriched in the CK group (red) and ET group (green). **(B)** Discriminative biomarkers with an LDA score>4 between the CK group (red) and ET group (green). **(C)** LEfSe cladogram represents taxa enriched in the ET group and ET-J group. **(D)** Discriminative biomarkers with an LDA score>4 between the ET group (red) and ET-J group (green). **(E)** Discriminative biomarkers with an LDA score>4 between the ET group (red) and ET-J group (green).

To find the specific main flora between each group, LEFse was used to analyze the abundance distribution of the three groups of intestinal flora (Figures 6A–H). As shown in Figures 6A,B, compared with the CK group, *Ruminococcaceae*, *Rikenellaceae*, *Clostridia*, *Allprevotella*, and *Proteobacteria* increased, while *Bacteroidales*, *Lachnospiraceae*, *Lactobacillus*, and *Prevotellaceae* decreased in the ET group. Mutlu et al. (2012) found that compared with healthy individuals, the abundance of *Enterobacteriaceae* and *Proteobacteria* was increased in patients with early ALD, while the number of *Bacteroidetes* and *Firmicutes* decreased. This is similar to the results of our research. The abundance of *Ruminococcaceae*, *Rikenellaceae*, *Alistipes*, and *Alloprevotella* in the ET group was significantly higher than that of the other groups (Figure 6E). For the ET-J, the abundance of *Firmicutes*, *Lachnospiraceae*, *Clostridia*, *Lactobacillales*, and *Proteobacteria* was significantly higher than that in the CK group. The fermentation broth adjusted the abnormally high content of *Ruminococcaceae*, *Rikenellaceae*, and *Alloprevotella* in the ET group and increased the abundance of *Lactobacillales*. These results indicate that oral intake of the fermentation broth can effectively improve the diversity of intestinal flora and restore the balance of intestinal flora, while alcohol can disturb the balance of intestinal flora.

## Discussion

Fermentation process is a useful tool to eliminate herbal medicine’s toxicity and improve its bioavailability. Therefore, the research on the fermentation of various traditional Chinese medicines increased rapidly. For example, Kwon et al. (2018) used *Lactobacillus paracasei* to ferment *P. lobata* extract. Gasinski et al. (2020) used *Saccharomyces cerevisiae* var. *diastaticus* to ferment the beer with *C. pinnatifida* fruit or *C. pinnatifida* juice. In this study, the *L. rhamnose* 217-1 was used to ferment three types of food and drug homologous raw materials, including *P. lobata*, *L. japonica*, and *C. pinnatifida*. The obtained product had a higher content of polyphenols, puerarin, and total isoflavones. It should be pointed out that the concentration of bioactive substances varied in different studies (Huang et al., 2017; Zhang et al., 2017). The inconsistency of the results may be caused by the use of different strains and the pretreatment of the substrate.

Polyphenols as natural antioxidants in plants can terminate the chain reaction by removing free radical intermediates and inhibiting other oxidation reactions (Nickavar et al., 2007). It has been reported that the *L. japonica* extracts have the strongest antioxidant capacity, which resulted from the isochlorogenic acid A, isochlorogenic acid B, isochlorogenic acid C, and other phenols (Liu et al., 2019). The pharmacological mechanism of puerarin on ALD may be related to the inhibition of alcohol-induced endotoxin secretion, keratinocyte activation, and endotoxin receptor protein expression (Peng et al., 2013). In addition, studies have shown that isoflavones and their metabolites can effectively scavenge peroxy radicals and inhibit lipid peroxidation through different mechanisms (Corinna and Sabine, 2006). Therefore, the increased concentration of polyphenols, puerarin, and isoflavones in the fermentation broth can improve the ability of free radical scavenging and reduce the liver damage caused by alcohol.

Amino acids participate in various biological activities. ALD usually led to the disorder of amino acid metabolism, which further caused malnutrition of the body (Chao et al., 2016). In turn, malnutrition increased the sensitivity of hepatocytes to alcohol toxicity (Patek, 1979; Mendenhall et al., 1984). Therefore, a diet containing appropriate amounts of amino acids could play a positive role in the treatment of liver diseases. Many studies on ALD treatment with amino acids have been reported (Tedesco et al., 2018). The underlying mechanism might be that the amino acids regulate the metabolic disorder caused by alcohol, stabilize the redox potential in the cytoplasm, accelerate the tricarboxylic acid cycle, and promote acetaldehyde decomposition to reduce liver tissue damage. In the present study, the obtained fermentation broth can meet ALD patients’ amino acid needs and thereby is a beneficial supplement of nitrogen sources for daily diet.

The fermentation using LAB in this study increased the concentration of polyphenols, puerarin, isoflavones, and amino acids in the fermentation broth. As mentioned above, amino acids could promote the body’s catabolism after ingesting alcohol. Polyphenols and isoflavones in the fermentation broth effectively removed the peroxy free radicals produced in the process of alcohol metabolism. Puerarin inhibited alcohol-induced endotoxin secretion. These substances together improved the tolerance of hepatocytes to alcohol toxicity, reduced the damage to the body after alcohol intake, especially the oxidative damage to the liver, and inhibited the liver inflammation induced by LPS secreted by alcohol, thus to prevent and alleviate ALD.

So far, extensive studies in rats and humans have shown that ALD is usually associated with increased oxidative stress and free radical-mediated tissue damage (Kim et al., 2014; Li et al., 2016). A feature of ALD is the release of liver enzymes to circulatory system such as ALT and AST. Therefore, serum ALT and AST levels can be the indicator for early ALD (Cao et al., 2015). In this study, we established the ALD mouse model to investigate the protective effect of the fermentation broth on the liver. It was found that the liver index significantly decreased, and the ALT and AST levels were strikingly elevated in the mice of the ET group. These results were consistent with the previous studies (Xu et al., 2018; Li et al., 2021). Furthermore, the treatment of fermentation broth significantly decreased the elevation of ALT and AST induced by alcohol, indicating that the fermentation broth could reduce alcohol-induced liver damage.

Glutathione and SOD could protect hepatocytes from ROS damage by scavenging lipid peroxides and oxygen free radicals. However, alcohol can inhibit GSH concentration and SOD activity, thus increasing oxidative stress. Therefore, they can directly reflect the liver’s antioxidant capacity (Glade and Meguid, 2017). In a previous research, the SOD concentration in the liver of ALD mice was increased to 17.5ng/g·port by using the fermentation broth of *L. plantarum* (Ran et al., 2020). While the higher SOD concentration of ET-J group mice was detected in our study. Besides, the GSH concentration was dramatically increased to 16.195±2.5mg/g·prot in the liver. This result was 2.45 times higher than that obtained by Xiang et al. (2012). In conclusion, our fermentation broth can improve the concentration of GSH and SOD, thus enhancing the antioxidant capacity of liver tissues and reducing oxidative stress.

More and more evidence showed that liver inflammation caused by intestinal LPS played a key role in ALD (Sung et al., 2016). The intestinal permeability gradually increased under the long-term action of alcohol. Therefore, the enteric bacteria translocated into the blood and lysed to produce LPS, causing a series of diseases such as endotoxemia. Furthermore, LPS directly entered the blood through the intestinal barrier, leading to the immune response of macrophages in the liver and triggering an inflammatory signaling mechanism. At last, the low-grade inflammation was produced and the inflammatory factors were released (Pohlmann et al., 2018). In the present study, LPS concentration was reduced and the inflammation factors were decreased significantly (*p*<0.01) in the ALD mice by feeding the fermentation broth of *P. lobata*, *L. japonica*, and *C. pinnatifida*, indicating the protective role in the body caused by ALD or further inflammation.

Studies have shown that probiotics (e.g., *Lactobacillus*) can change the composition of the intestinal flora, thereby preventing alcohol-induced malnutrition, intestinal permeability, bacterial translocation, endotoxemia, and the development of ALD (Engen et al., 2015). At present, two mechanisms of action of *Lactobacillus* on ALD are widely accepted. One is that *Lactobacillus* can restore the imbalance of intestinal flora caused by ALD. The second mechanism is the immune regulation of *lactobacilli* on the immune response system of intestinal-related lymphatic and epithelial cells. The introduction of *Lactobacillus* can produce protection by mediating T cells and regulating the balance between Th1 and Th2 by stimulating and producing different cytokines. However, the interaction between *Lactobacillus* and the immune system remains to be clarified (Bao and Yang, 2018).

The symbiotic relationship between the gut flora and the liver is regulated and stabilized through a complex network of interactions (Kho and Lal, 2018). The gut-liver axis has an impact on the pathogenesis of many chronic liver diseases (including ALD). The study of Posteraro et al. (2018) found evidence and clues for the role of the gut-liver axis. Their study showed that the EtOH-induced early liver disease model in rats indicated that changes in the intestinal flora might contribute to EtOH-induced intestinal and liver pathological changes (such as inflammation and liver pathology). Alcohol and its degradation products destroy the epithelial TJ, leading to increased intestinal permeability and inflammation (Cassard and Ciocan, 2017). Intestinal PAMPs (such as endotoxin) increase after heavy drinking (Szabo, 2015). Generally, increased intestinal permeability and bacterial translocation may allow microbial metabolites to reach the liver, which can impair the metabolism of bile acid (BA) and promote abnormal intestinal motility and systemic inflammation. All of these conditions may cause enteral malnutrition, which further increases liver damage (Milosevic et al., 2019). Active ingredients in fermentation broth (e.g., puerarin) can reduce the damage of gastrointestinal mucosa and prevent the increase of intestinal permeability by regulating the intestinal epithelial barrier, inhibiting the gastrointestinal absorption of alcohol, and accelerating the degradation and metabolism of alcohol. Simultaneously, probiotics can prevent the increase of Gram-negative bacteria and restore the balance of the intestinal flora. The combined effect of these two aspects prevents the increase of LPS content in the blood, inhibits the transmission of inflammatory signals in the intestine-liver axis, and weakens the liver’s immune response, thereby reducing the levels of IL-6 and TNF-α and alleviating inflammation reaction.

## Conclusion

The fermentation of LAB significantly increased the concentration of polyphenols, puerarin, isoflavones, and amino acids in the fermentation broth of *P. lobata*, *L. japonica*, and *C. pinnatifida*. Animal experiments showed that the fermentation broth effectively inhibited ALT and AST activity, improved the concentration of GSH and SOD, and decreased the concentration of LPS, IL-6, and TNF-ɑ. Furthermore, supplementing the fermentation broth increased the abundance of *Lactobacillus* in the intestines of chronic ALD mice. These results indicated that the fermentation broth could protect the liver by reducing liver damage caused by alcohol-induced oxidative stress, inflammation, and intestinal flora disturbance. It should be noted that only the early stages of liver damage were investigated in the present study, and the therapeutic effect of the fermentation broth on the middle and late stages of liver damage and its clinical effect needs further research. In addition, the influence of fermentation broth on the permeability of the intestinal epithelium is also worthy of studying.

## Data Availability Statement

The raw data supporting the conclusions of this article will be made available by the authors, without undue reservation.

## Ethics Statement

The animal study was reviewed and approved by Experimental Animal Welfare Ethics Committee of Yantai Raphael Biotechnology Co., Ltd.

## Author Contributions

ZX and XL: conceptualization and supervision. ZY, XC, and LY: methodology. ZW: software. TW, ZX, and XL: validation and funding acquisition. TW, ZW, and ZY: writing – original draft preparation. TW, ZW, ZY, ZX, and XL: writing – review and editing. All authors contributed to the article and approved the submitted version.

## Conflict of Interest

The authors declare that the research was conducted in the absence of any commercial or financial relationships that could be construed as a potential conflict of interest.

## Publisher’s Note

All claims expressed in this article are solely those of the authors and do not necessarily represent those of their affiliated organizations, or those of the publisher, the editors and the reviewers. Any product that may be evaluated in this article, or claim that may be made by its manufacturer, is not guaranteed or endorsed by the publisher.
